# Effect of multiorgan abdominal ischemic preconditioning on experimental kidney transplantation

**DOI:** 10.1590/acb400225

**Published:** 2024-12-20

**Authors:** Juan Cruz Abate, Ivana Ivanoff Marinoff, Nathalie Arnal, Mariana Machuca, Rodrigo Papa-Gobbi, Leandro Vecchio, Martín Rumbo, Pablo Stringa, Natalia Raquel Lausada

**Affiliations:** 1 Universidad Nacional de La Plata – Faculty of Medicine – Organ Transplant Laboratory – La Plata – Argentina.; 2 Universidad Nacional de La Plata – Institute for Immunological and Pathophysiological Studies – Faculty of Exact Sciences – La Plata – Argentina.; 3 Biochemistry Research Institute of La Plata – Faculty of Medicine – Neuroscience Laboratory – La Plata – Argentina.; 4 Universidad Nacional de La Plata – Faculty of Veterinary Sciences – Special Pathology Laboratory – La Plata – Argentina.

**Keywords:** Reperfusion Injury, Kidney Transplantation, Warm Ischemia, Ischemic Preconditioning, Models, Animal

## Abstract

**Purpose:**

To mitigate ischemia-reperfusion injury (IRI) triggered in solid organ transplant procedures, we aimed to evaluate the effects of multi-organ abdominal ischemic preconditioning (MAIP) in the context of renal IRI.

**Methods:**

An experimental kidney transplant model was conducted. Rats were divided into three groups: an intervention free basal group from which physiological data was collected; a control group (CT), which consisted of transplanted animals without MAIP; and a treated group, in which a MAIP protocol was implemented in the donor during the procurement of the left kidney, monitoring the recipient for 24 hours.

**Results:**

Urea, creatinine, and lactate dehydrogenase, as well as histopathological analysis (Banff: CT 1,66 ± 0,57 vs. basal 0, and MAIP 1), showed a clear trend in favor of MAIP group. Similar results were observed for tumor necrosis factor-α, interleukin-6 and CXCL10, as well as indicators of oxidative stress, with statistically significant levels for CXCL10 [0,295 ± 0,0074 arbitrary units (AU) CT and 0,0057 ± 0,0065 AU MAIP] and TBARS (2,93 ± 0,08 nmol/μg CT; and 2,49 ± 0,23 nmol/μg MAIP; *p* 0.05).

**Conclusion:**

The findings indicated that the MAIP exerts a protective influence on the transplanted kidneys, functioning as an IRI-protective strategy and enhancing the parameters associated with renal graft functionality.

## Introduction

Ischemia-reperfusion injury (IRI) is an inherent component of solid organ transplantation procedure. It plays a significant role in early allograft dysfunction, leading to a higher incidence of graft rejection and reduced long-term allograft survival. Additionally, it is recognized as the primary cause of acute kidney injury (AKI) on a global scale. AKI frequently happens in the context of multiple-organ failure, sepsis, vascular occlusion and kidney transplantation, with IRI being the underlying mechanism^1^^-^ 3.

IRI is a dynamic process comprising two distinctive yet interrelated phases: ischemic damage, and inflammation-mediated reperfusion injury. The deleterious effects on organs result from various cellular and molecular pathways, involving the production of oxygen and nitrogen reperfusion free radicals, as well as the activation of the innate immune response and the complement system^4^.

Numerous strategies have been proposed to mitigate IRI in the context of organ transplantation. However, most of these remain in experimental phases with only a few currently implemented in clinical practice. One such approach is ischemic preconditioning (IPC), which involves exposing the target organ to brief periods of ischemia and subsequent reperfusion. A notorious protective effect against IRI can be seen as a consequence. Several experimental studies have been performed to evaluate the effect of IPC in organ transplantation. Although the precise mechanism remains incompletely understood, these studies consistently demonstrate that IPC confers protection against IRI in the kidney, liver and visceral transplantation, reducing the inflammatory response and the oxidative and nitrative stress^5^^-^ 7.

The evidence accumulated through the application of the IPC has been applied with a focus on the nutrient vasculature of a particular organ. For instance, intestinal IPC involves clamping the superior mesenteric artery, hepatic IPC employs the Pringles maneuver, and renal IPC relies on renal artery occlusion. However, an IPC procedure that encompasses all abdominal organs has not been considered yet. Taking into account the practical context of multi-organ abdominal procurement for transplantation, we aimed to evaluate the effects of multi-organ abdominal ischemic preconditioning (MAIP) in the context of renal IRI^8^^-^ 10.

## Methods

Male, adults Sprague-Dawley (SD) rats (weight 283 ± 37 g) were used. Experimental protocols were approved by the Institutional Committee for the Care and Use of Laboratory Animals of the Faculty of Medicine, Universidad Nacional de La Plata (Protocol Numbers: T05-02-2016). The animals were handled in strict adherence to both local and international ethical standards governing the humane treatment of vertebrate animals employed in biomedical research.

Surgical procedures were performed using inhalation anesthesia (5% isoflurane for induction, 2% for maintenance, oxygen 0,5–1 L/min). Tramadol (30 mg/kg) was administered subcutaneously every 12 hours as an analgesic, and ceftriaxone (65 mg/kg/24 h) as a post-surgical antibiotic therapy.

### Step 1: Multi-organ abdominal ischemic preconditioning associated with ischemia-reperfusion injury

**Figure 1:**
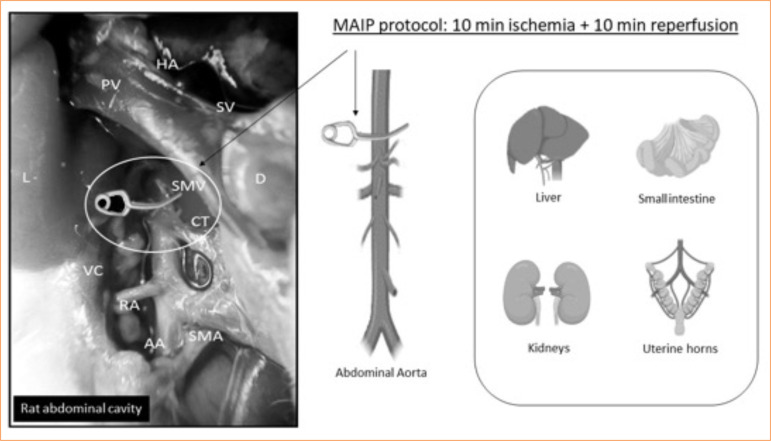
The multiorgan abdominal ischemic preconditioning (MAIP) protocol is executed by clamping the supraceliac abdominal aorta in rats. The primary structures involved include the portal vein, hepatic artery, splenic vein superior mesenteric vein, duodenum, celiac trunk, vena cava, renal artery, abdominal aorta, and superior mesenteric artery.

Source: Elaborated by the authors.

In the first step, we evaluated the effect of a MAIP protocol associated with a prolonged period of ischemia and subsequent reperfusion. Three experimental groups of SD rats were established, each group with n = 6 animals:

- Basal: This group was included for physiological data collection and underwent no interventions;
- Control (CT): Bilateral renal ischemia was generated by clamping the renal arteries of both kidneys simultaneously for 40 min, followed by 24 hours of reperfusion;
- MAIP: Prior to the prolonged ischemia and 24 hours of reperfusion period, as in the CT group, a multi-organ ischemic preconditioning protocol was implemented. This involved transient clamping of the abdominal aorta artery for 10 min, followed by 10 min of reperfusion (Fig. 1).

### Step 2: MAIP associated with experimental kidney transplantation

An experimental model of kidney transplantation already described by us and other research groups was carried out^11^^,^ 12. In summary, the donor’s left kidney was perfused *in situ* with 3 mL of cold (4°C) heparinized Ringer Lactate solution via the infrarenal aorta until it was macroscopically bloodless. Subsequently, the kidney was extracted, including the renal artery attached to a segment of the aorta, and the renal vein was excised at its origin. The graft was then stored in the same solution and transplanted into the abdominal cavity of the recipient. The donor’s aorta and renal vein were then respectively anastomosed end-to-side to the recipient’s aorta and inferior vena cava using 9-0 nonabsorbable monofilament nylon suture. An end-to-end ureter anastomosis was performed using a 10-0 nonabsorbable monofilament nylon suture. The right native kidney of the recipient was excised at the time of transplantation.

Fifteen SD rats were divided into three groups (n = 5 per group). One was the basal, with rats included for physiological data collection and no interventions; and two transplant groups:

- CT, in which isolated heterotopic kidney transplantation without treatment was performed;
- MAIP, that included a multiorgan preconditioning applied to the donor (10 min of ischemia followed by 10 min of reperfusion) before the extraction of the left kidney, which was preserved at 4°C for 40 min in a preservation solution (Ringer Lactate) and then implanted in the recipient animal the same way as in the CT group.

Recipient animals underwent bilateral nephrectomy prior to renal transplantation.

### Sampling and analysis

In both experimental steps (transient vascular occlusion and kidney transplantation), the animals were sampled and euthanized 24 hours after renal reperfusion.

Kidney samples were fixed in 10% formalin for approximately 48 hours, then dehydrated with 70% alcohol and stained with hematoxylin-eosin (H-E). Histopathological damage was assessed according to the Banff score^13^^-^ 15. At the same time, blood was obtained to determine urea, creatinine and plasma lactate dehydrogenase (LDH) values.

Also, tissue samples were processed for gene expression and oxidative stress analysis. Realtime polymerase chain reaction was performed following manufacturer’s protocol using the iCycler thermal cycler (BioRad, Philadelphia, PA, United States of America). Primers for rat interleukin-6 (IL-6), tumor necrosis factor-α (TNF-α), CXCL10, and Actin-b were designed by us or adapted from literature^16^.

The biochemical determination of damage due to nitrative and oxidative stress that considers thiobarbituric acid reactive substances (TBARs); protein carbonyls (CPs), and the ferric reducing activity of plasma (FRAP) were evaluated. The set of data obtained from the analysis variables was represented in a multidimensional graph^17^^-^ 23.

The statistical analyses were performed using GraphPad 8.00 (GraphPad Software, San Diego, California, United States of America).

Differences between groups were considered statistically significant when *p* < 0.05.

## Results

**Figure 2:**
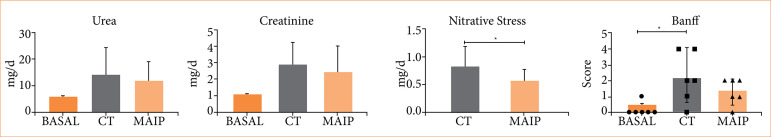
Results of urea, creatinine, nitrative stress, and histopathological scores in the CT and MAIP animals following prolonged renal ischemia. The most significant differences between the CT group and the treated group were observed in the histological analysis and the levels of nitrosative stress.

MAIP: multi-organ abdominal ischemic preconditioning; CT: control group; *p < 0.05. Source: Elaborated by the authors.

The MAIP treatment, when associated with a period of prolonged ischemia and subsequent reperfusion, yielded intriguing and encouraging outcomes regarding renal protection against IRI. These outcomes were statistically significant regarding nitrative stress and histological features of 24-hour reperfused kidneys (Fig. 2). As main histopathological alterations, CT group samples exhibited signs of acute tubular necrosis, whereas MAIP group showed histological signs of hydropic degeneration.

**Figure 3:**
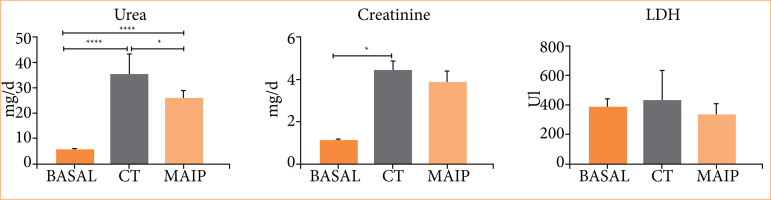
Results of urea, creatinine, and lactate dehydrogenase in the basal, CT, and MAIP groups associated with experimental kidney transplantation in rats. The MAIP group demonstrated a statistically significant decrease in urea levels compared to the CT group.

MAIP: multi-organ abdominal ischemic preconditioning; CT: control group: LDH: lactate dehydrogenase; * *p* < 0.05; **** *p* < 0.0001. Source: Elaborated by the authors.

The application of MAIP in the context of kidney transplantation was observed to result in a reduction in urea levels when compared to those of CT recipients. Additionally, a clear trend in favor of the MAIP group was evident in the analysis of creatinine and LDH values (Fig. 3). Also, beneficial results of MAIP treatment were observed in the gene expression analysis.

**Figure 4:**
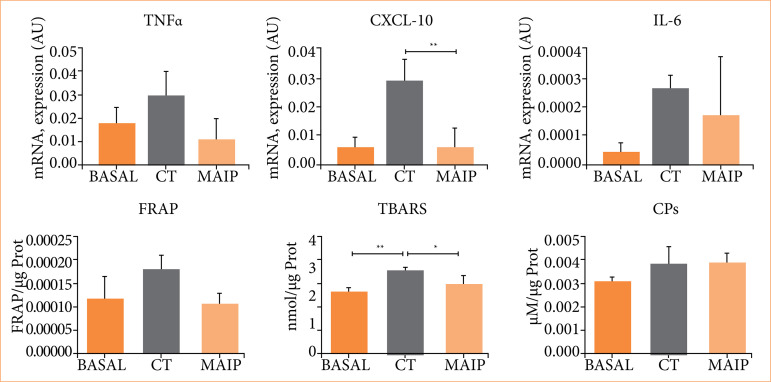
Determinations of mRNA (TNF-α, CXCL10, IL-6) and indicators of oxidative stress, including FRAP, TBARs, and CPs, in the basal, CT, and MAIP groups associated with kidney transplantation.

MAIP: multi-organ abdominal ischemic preconditioning; CT: control group; TNF: tumor necrosis factor; IL-6: interleukin-6; FRAP: ferric reducing activity of plasma; TBARS: thiobarbituric acid reactive substances; CPs: protein carbonyls; *p < 0.05; **p < 0.01. Source: Elaborated by the authors.

Despite increased TNF-a and IL-6 in CT recipients relative to baseline and MAIP animals, statistical differences were observed between treated and untreated donors in CXCL10 measurements (Fig. 4). On the other hand, oxidative stress indicators (FRAP, TBARS, and CPs) have presented similar values between CT and MAIP experimental transplant groups. A decrease in TBARS stands out in the MAIP group with statistical significance (p < 0.05) between CT group vs. baseline, and CT group vs. MAIP (Fig. 4).

**Figure 5:**
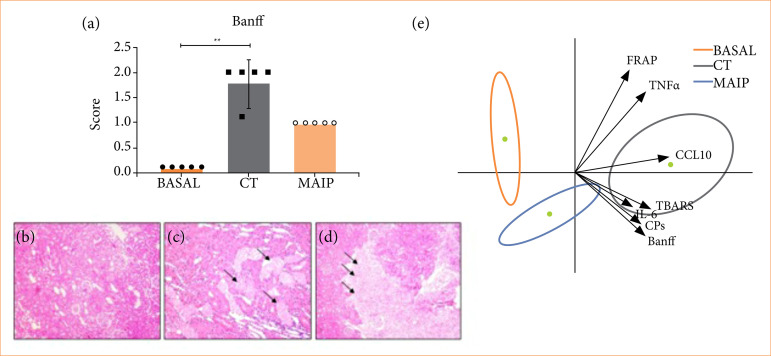
The Banff score in CT, and MAIP groups associated with kidney transplantation is presented. **(a)** Representative images of kidneys (hematoxylin and eosin 20x and 40x) demonstrate **(b)** the absence of lesions, **(c)** focal tubular necrosis, and **(d)** hemorrhage as evaluated parameters. Considering all assessed variables, **(e)** principal component analysis reveals the protective effects of MAIP against renal IRI.

MAIP: multi-organ abdominal ischemic preconditioning; CT: control group; TNF: tumor necrosis factor; IL-6: interleukin-6; FRAP: ferric reducing activity of plasma; TBARS: thiobarbituric acid reactive substances; CPs: protein carbonyls; **p < 0.01 CT vs. baseline. Source: Elaborated by the authors.

The histopathological analysis with H-E staining shows an increase in the Banff score in the CT group (1.66 ± 0.57) in relation to the Baseline (score = 0), with a lower score in MAIP (score = 1) in relation to CT (Fig. 5). The principal component analysis revealed that each individual was grouped according to their respective group characteristics (baseline vs CT vs MAIP). The only variable that differs from the group is FRAP, which despite being a protection marker presents a result that is related to the CT group. Although statistically significant data is not obtained in all the variables, there are clear signs of damage in the CT graft, and individual improvement of the parameters in the MAIP transplanted animals (Fig. 5).

## Discussion

Since the advent of transplantation as a therapy for terminal organ or tissue failure –often celebrated as one of the miracles of the 20th century–, substantial advancements have been made in this field. However, the persistently high number of patients on waiting lists remains alarming, as the available organs are insufficient to address this critical disparity. Consequently, efforts to enhance effective organ donation provide essential benefits for patients.

This experimental study aligns with the principles of health research and aims to elucidate strategies for improving the quality of abdominal organs through increased procurement. IRI is inherent to all transplant procedures, as highlighted in the introduction of this research. The detrimental effects of IRI on the functionality of transplanted solid organs have driven investigations into the underlying biological processes associated with graft procurement and implantation, with the goal of implementing strategies to mitigate damage caused by transplant-related IRI. Approaches such as preservation solutions, hypothermia, and various pharmacological agents exemplify these strategies^4^.

In this context, IPC has been explored as a surgical strategy to attenuate IRI, with its effects assessed across various abdominal solid organs. Encouraging findings indicate that IPC confers protective benefits against IRI. However, most studies have focused on preconditioning individual organs, specifically targeting their vascular pedicles. Organ-targeted IPC has demonstrated the ability to reduce liver enzymes in the post-transplant period, enhance renal functionality following graft reperfusion, and preserve intestinal and uterine architecture after an IRI event^5^^-^ 10 ^,^ 24. This evidence has inspired the challenge of developing a preconditioning protocol that impacts multiple abdominal organs, as demonstrated in the present study.

MAIP represents an innovative surgical technique aimed at standardizing treatment across a range of intra-abdominal organs, thereby facilitating its application during multiorgan extraction in clinical practice. Our results indicate that MAIP is well tolerated in individuals subjected to 10 min of ischemia followed by 10 min of reperfusion at the origin of the abdominal aorta. As a strategy for IRI mitigation, MAIP has yielded promising results across the liver, small intestine, kidneys, and uterus, corroborating findings from other researchers who have examined IPC on an organ-specific basis^5^^-^ 10 ^,^ 24.

Our findings demonstrate a clear renal benefit, supported by objective serological and histological data indicating organ protection. These results are consistent with those of Brown et al.^2^, who reported significant renal protection from IPC, evidenced by serum levels of urea, creatinine, acute tubular necrosis, and fibrosis in both acute and chronic phases following an IRI event. Interestingly, the protocol evaluated by this group consisted of multiple ischemic events followed by reperfusion prior to a prolonged ischemic period, suggesting, alongside other literature, that various effective IPC protocols exist to enhance renal functionality post-reperfusion.

In comparison with recent studies evaluating pharmacological pre-treatments in experimental kidney transplantation models, our findings regarding post-IRI parameters are similar. Zou et al.^25^ demonstrated that donor pre-treatment with the glucose derivative 3-O-methyl-D-glucose (OMG) improved renal functions after kidney transplantation, as evidenced by reduced levels of urea, creatinine, pro-inflammatory cytokines, and favorable renal histology. Additionally, Nelson et al.^26^ recently assessed the effects of sodium thiosulfate (STS) in a renal transplantation model in rats. Although no significant differences were found in several evaluated parameters post-transplant, the authors concluded that STS pre-treatment may lead to improved graft outcomes and prolonged recipient survival, particularly when combined with the University of Wisconsin preservation solution. Our results show similarities with these studies, highlighting the capability of MAIP to attenuate oxidative and nitrosative stress following an IRI event, reduce the expression of pro-inflammatory genes such as CXCL-10, and trend toward improvements in various evaluated parameters, aligning with findings by Nelson et al.^26^.

Notably, MAIP offers advantages over pharmacological pre-treatments; it incurs no additional cost compared to drug strategies. Furthermore, when a drug is administered to a donor, it disseminates throughout the entire body, affecting all organs and tissues without the possibility of targeting specific areas^11^. In contrast, both IPC and MAIP allow for the selection of organs to be treated in the donor, whether a single organ (IPC) or multiple organs, as proposed in this manuscript. This specificity enables medical teams to devise tailored strategies based on the organs to be transplanted.

Despite the promising results of MAIP concerning renal protection, a limitation of our study is the lack of comprehensive evaluation across other transplant procedures. Therefore, further experimental evidence is necessary to assess the effects of MAIP on additional transplantable abdominal organs, such as the liver and small bowel. Addressing this challenge would bolster the case for IPC pre-treatment in the procurement of multiple organs for transplantation. Nevertheless, this study contributes to demonstrating that the application of MAIP as a novel surgical strategy in isolated heterotopic kidney donors in rats affirms its efficacy in mitigating IRI, as evidenced by improvements in parameters associated with renal graft function.

## Conclusion

MAIP emerges as a promising multi-organ abdominal treatment that protects the kidney against IRI, as we have shown in the present study.

## Data Availability

The data will be available upon request.
